# Supplementary material for: Amino Acid and Glucose Fermentation Maintain ATP Content in Mouse and Human Malignant Glioma Cells
Source: ASN Neuro. 2024 Dec 2;16(1):2422268. doi: 10.1080/17590914.2024.2422268 (PMC11792161; doi:10.1080/17590914.2024.2422268)
Supplement: Supplemental Material [file TASN_A_2422268_SM8536.docx]

**Supplemental Figure 1**


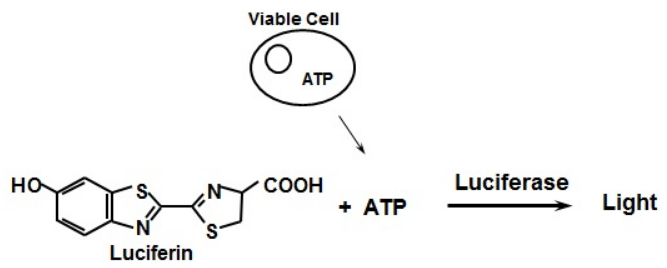

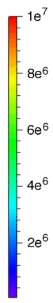


**A**

**B**

**Supplemental Figure 1.** **Correlation between seeding density and bioluminescence.** A) Schematic of ATP-dependent luciferin-luciferase bioluminescent reaction. Modified from *Cell Viability Assays*. B) VM-M3 cells were seeded in seeding media and adhered for 6 hours. Media was swapped from seeding media to experimental media (without phenol red and serum) immediately prior to bioluminescence reading. Luciferin (10 mg/mL) was applied, and the reading was taken after five minutes. 4-5 measurements in 96-well plates were used for each seeding density. Pearson’s correlation analysis was performed with a 95% confidence interval (0.9261-0.9985).

**Supplemental Figure 2**

**Supplemental Figure 2. Influence of glucose and glutamine on cell viability.** A) VM-M3, C) CT-2A, and E) U-87MG cells were seeded at a density of 5.0 x 10^3^ and cultured for 24 hours with indicated media compositions. Glucose was added at 12 mM and glutamine (Q) was added at 2 mM. BM represents DMEM with no added glucose or glutamine. Trypan blue exclusion assay was used to determine viability. B) VM-M3, D) CT-2A, and F) U-87MG cells were cultured as described above. Calcein-AM and ethidium homodimer III (EthD-III) staining assay was used to determine viability. Data are shown as mean ± SEM with three independent experiments.

**A**

**B**

**C**

**D**

**E**

**F**

**Supplemental Figure 3**

**B**

**PKM1**

**PKM2**

**Actin**

**Actin**

**A**


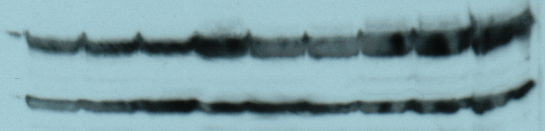

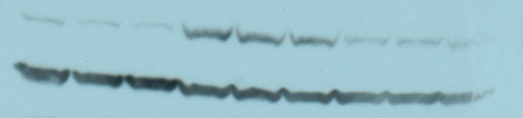


**VM-M3 CT-2A U-87MG**

**A**

**Supplemental Figure 3. Presence of PKM2 in VM-M3, CT-2A, and U-87MG cells.** A) Representative western blots of PKM1 and PKM2 expression in VM-M3, CT-2A, and U-87MG cells. Actin was used as a loading control. B) Extracellular lactate produced by glucose, glutamine, or both after 6 hours. Lactate was measured by colorimetric enzymatic assay. Background reading (basal media) was subtracted from all values. Bars represent three independent experiments.

**Supplemental Figure 4**

**A**

**B**

**Supplemental Figure 4. Influence of supplemented amino acids on bioluminescence in basal media.** A) CT-2A and B) U-87MG cells cultured in basal media with the indicated amino acids supplemented at 4 mM. Amino acids are ordered by descending percent change at hour 24. Cells were seeded at a density of 1.0 x 10^4^ cells/well. The bioluminescence value at hour 0 is represented by a dashed line. Values are measured as mean ± SEM with three or four independent experiments.

**Supplemental Figure 5**

**Supplemental Figure 5. Influence of Potassium Cyanide on Complex IV Activity.** VM-M3 cells were cultured with (blue) or without (black) the addition of 1 mM KCN. The absorbance was measured at 550 nm for 20 minutes upon the addition of 50 µM ferrocytochrome C. Values are shown as mean ± SD with three independent experiments.

**Supplemental Table 1**

| Amino Acid | Source (Catalog) |
| --- | --- |
| Alanine | Thermo Fisher Scientific (56-41-7) |
| Arginine | Thermo Fisher Scientific (74-79-3) |
| Asparagine | Alfa Aesar (A15012) |
| Aspartic Acid | Sigma-Aldrich (A6683) |
| Cysteine | Alfa Aesar (J63745.14) |
| Glutamic Acid | Sigma-Aldrich (49621) |
| Glutamine | Sigma-Aldrich (G8540) |
| Glycine | Sigma-Aldrich (410225) |
| Histidine | Sigma-Aldrich (H-8125) |
| Isoleucine | Sigma-Aldrich (I2752) |
| Leucine | Alfa Aesar (J62824) |
| Lysine | Alfa Aesar (A16249) |
| Methionine | Sigma-Aldrich (M9625) |
| Phenylalanine | Alfa Aesar (A12328) |
| Proline | Thermo Fisher Scientific (A10199) |
| Serine | Alfa Aesar (J62187) |
| Threonine | Acros Organics (72-19-5) |
| Tryptophan | Thermo Fisher Scientific (J62508) |
| Tyrosine | Alfa Aesar (J63511) |
| Valine | Alfa Aesar (J62943) |

**Supplemental Table 1** – Amino acids and their sources used in this study.
